# Feasibility and Preliminary Efficacy of a “Sit Less” Program Leveraging Fitbit Tracking and Tailored Text Messages in Cardiometabolic Disease: Findings From 2 Parallel Randomized Controlled Trials in Coronary Artery Disease and Type 2 Diabetes

**DOI:** 10.2196/56497

**Published:** 2026-08-25

**Authors:** Chorong Park, Mary S Dietrich, Britta Larsen, Lindsay S Mayberry, Abigail Doyle, Soojung Ahn, Jason Jean, Kevin Maquiling, Mulubrhan F Mogos, James M Muchira, Shelagh Mulvaney

**Affiliations:** 1 College of Nursing, Seoul National University, 103 Daehak-ro, Jongno-gu, Main Nursing Building #501, Seoul, 03080, Republic of Korea, 82 27408827; 2 The Research Institute of Nursing Science, Seoul National University, Seoul, Seoul, Republic of Korea; 3 School of Transdisciplinary Innovations, Seoul National University, Seoul, Republic of Korea; 4 School of Nursing, Vanderbilt University, Nashville, TN, United States; 5 Department of Biostatistics, School of Medicine, Vanderbilt University, Nashville, TN, United States; 6 Herbert Wertheim School of Public Health and Human Longevity Science, University of California San Diego, La Jolla, CA, United States; 7 Division of General Internal Medicine and Public Health, Department of Medicine, Vanderbilt University Medical Center, Nashville, TN, United States; 8 Vanderbilt Institute for Clinical and Translational Research, Vanderbilt University School of Medicine, Nashville, TN, United States; 9 William F. Connell School of Nursing, Boston College, Chestnut Hill, MA, United States; 10 Vanderbilt University Medical Center, Nashville, TN, United States; 11 Department of Biomedical Informatics, Vanderbilt University, Nashville, TN, United States; 12 College of Connected Computing, Vanderbilt University, Nashville, TN, United States

**Keywords:** sedentary behavior, mobile health, mHealth, digital health, activity tracker, cardiovascular disease, diabetes, text messages

## Abstract

**Background:**

Individuals with cardiometabolic disease typically average 10 to 14 hours of daily sedentary time, which increases the risk of cardiovascular disease.

**Objective:**

This study aimed to evaluate the feasibility, acceptability, and preliminary efficacy of a “Sit Less” program in reducing sedentary behavior in people with cardiometabolic disease.

**Methods:**

Participants with cardiovascular disease or type 2 diabetes underwent separate randomization into the Sit Less intervention or the control group. Sit Less included 1 in-person counseling session, a Fitbit, a smart water bottle, and tailored weekly text messages for 12 weeks. Control group participants received the American Heart Association’s “Answers by Heart” booklet. Sedentary behavior was measured using the activPAL for 7 days at baseline and postintervention. The secondary outcomes included physical activity, cardiometabolic biomarkers, continuous glucose monitoring metrics, and psychological factors. Generalized linear models estimated adjusted between-group differences, controlling for baseline values, cohort, cohort×group interaction, and leisure-time exercise.

**Results:**

Of the 37 randomized participants, 35 (95%) completed the study (Sit Less: n=17, 48.6%; and control: n=18, 51.4%). Of the 35 participants who completed the study, 62% (n=21) were men, the median age was 67 (54-72) years, and their baseline sedentary time was approximately 10.4 hours per day. Sit Less participants demonstrated high adherence, including a text response rate of 81% and a median Fitbit wear time of 15 hours per day on 6.8 days per week, and 15 (88%) of 17 participants reported satisfaction with the program. Compared with the control group, Sit Less participants showed greater reductions in total sedentary time (adjusted mean difference −17.3 min/d, 95% CI −63 to 28; *P*=.45) and prolonged sedentary time of >30 minutes (−39.5 min/d, 95% CI −100 to 21; *P*=.20) and >60 minutes (−49.9 min/d, 95% CI −102 to 2; *P*=.06) and increases in sit-to-stand transitions (4.2 per day, 95% CI −2 to 10; *P*=.13), although none were statistically significant. Hemoglobin A_1c_ was lower in the Sit Less group than in the control group (adjusted mean difference −0.4%, 95% CI −0.8 to −0.1; *P*=.04). Continuous glucose monitoring outcomes, including mean glucose (−6.0 mg/dL, 95% CI −14.9 to 2.8; *P*=.18) and time in range (2.1%, 95% CI −4.8 to 9.1; *P*=.54), were not statistically significant but demonstrated similar directional changes. Other cardiometabolic biomarkers, physical activity, and psychological outcomes were not significantly different, except for lower self-efficacy for moderate physical activity in the Sit Less group (−0.6, 95% CI −1.1 to −0.2; *P*=.003).

**Conclusions:**

The Sit Less intervention was feasible and acceptable and showed possible improvements in sedentary behavior and glycemic outcomes. Although this pilot study had a small sample size and limited statistical power, with multiple outcomes examined and wide CIs observed, these findings support evaluation in larger, adequately powered trials.

## Introduction

### Background

Sedentary behavior is a strong modifiable risk factor for cardiometabolic disease [1,2]. It involves a lack of leg muscle contractions and restricted blood flow behind the knee when sitting, which causes reduced insulin sensitivity, vascular dysfunction, activation of low-grade inflammatory responses, and an imbalance in energy expenditure [3]. As a result, greater total sedentary time is related to increased cardiometabolic risk [2], including decreased high-density lipoprotein levels and increased triglycerides, fasting glucose [4], BMI, and waist circumference [5]. Prolonged sedentary time is also associated with a 12% higher risk of incidental cardiovascular disease (CVD) independent of physical activity levels [1]. Emerging evidence suggests that physical activity attenuates cardiometabolic risk but does not eliminate the increased risk associated with high sedentary time [1,6]. Despite the important role of sedentary behavior in CVD and metabolic disease, patients with cardiometabolic diseases, including patients with coronary artery disease or diabetes, spend 70% to 90% of their waking time in sedentary behavior [7-9] and 50% of their total sedentary time is prolonged (>30 min per bout) [7]. The reason for this prolonged sedentary time among these individuals may relate to fear of moderate-to-vigorous levels of physical activity due to their heart condition among those with CVD or the perceived development of hypoglycemia among those with diabetes [10,11]. Targeting sedentary behavior, which occupies most of the waking time of patients with cardiometabolic disease, could be a promising target behavior for secondary prevention in this population.

Frequent standing or walking can reduce total sedentary time and disrupt prolonged sitting, changing physiological pathways and improving cardiometabolic outcomes [12-14]. Others have shown that reducing sedentary time with frequent breaks is safe in other chronic health conditions and applicable to patients with cardiometabolic diseases [15-17]. Mobile technologies, including activity trackers (ie, Fitbit) and smart water bottles, offer a low-cost and sustainable approach to reducing sedentary behavior. The use of wearable technologies may be a promising strategy to reduce total sedentary time and disrupt prolonged sitting patterns through features such as self-monitoring and prompts. In addition, the use of a smart water bottle can naturally break sedentary time by encouraging increased water intake, resulting in more frequent trips to the kitchen and restroom. To date, no wearable technology–based sedentary behavior reduction programs focusing on sedentary breaks for patients with cardiometabolic diseases are available.

### Study Objectives

This study aimed to assess the feasibility and acceptability of a multitechnology-based sedentary behavior reduction intervention (Sit Less program) in patients with cardiometabolic disease (CVD) or type 2 diabetes (T2D) and evaluate its preliminary efficacy in changes in sedentary behavior measured by total sedentary time, prolonged sedentary time (sedentary bouts >30 min and 60 min), and number of sit-to-stand transitions.

We hypothesized that participants in the Sit Less program would show greater reductions in total sedentary time and the number of prolonged sedentary bouts compared to those in the control group receiving usual care with an American Heart Association educational booklet. We also explored changes in secondary outcomes, including physical activity (eg, stepping time and standing time), cardiometabolic markers (eg, 24-h glycemic control, hemoglobin A_1c_ [HbA_1c_], BMI, waist-to-hip ratio, blood pressure, insulin, high-sensitivity C-reactive protein [hs-CRP], and lipids), and psychological factors (eg, confidence in reducing sedentary behavior, confidence in increasing light levels of physical activity and moderate-to-vigorous levels of physical activity, and habit strength for sedentary behavior).

## Methods

### Study Design

Our study included 2 cohorts of participants with different cardiometabolic diseases (CVD or type 2 diabetes), each completing a randomized (1:1 ratio) controlled trial. The same protocol was implemented in each cohort, with randomization occurring separately. Both studies were approved by the Vanderbilt Institutional Review Board and registered on ClinicalTrials.gov (CVD cohort: NCT05534256, August 24, 2022; T2D cohort: NCT05691452, January 5, 2023). A detailed description of the study protocol has been published elsewhere [18]. The study was conducted in accordance with the CONSORT (Consolidated Standards of Reporting Trials) guidelines (Checklist 1).

### Participants

Participants were eligible for inclusion in the trial if they were aged ≥18 years and had at least one of the following conditions: history of heart attack, coronary or carotid artery disease, ischemic heart disease, coronary stent placement, coronary artery bypass surgery, or T2D; self-reported sitting for ≥8 hours per day; ability to stand and walk; and owned a smartphone. Exclusion criteria included current use of an activity tracker, current participation in exercise or cardiac rehabilitation programs, non–English-speaking, unstable health conditions (eg, heart failure and uncontrolled arrhythmia) or kidney disease that limited daily water intake, any other conditions contradictory to standing or walking due to physical or cognitive limitations, or current pregnancy.

This pilot study was designed to assess the feasibility and acceptability of the Sit Less program rather than to conduct a fully powered hypothesis test. According to Viechtbauer et al [19], to detect a feasibility issue that has a 10% likelihood of occurrence, a sample size of 30 participants is required to identify the feasibility issue with a 95% CI.

### Ethical Considerations

This study was approved by the Institutional Review Board of Vanderbilt University Medical Center (IRB: 220416 and 221566) and conducted in accordance with the Declaration of Helsinki. All participants provided written informed consent and were informed of their right to withdraw at any time. Data were deidentified to ensure confidentiality. All participants received a possible total of US $150—US $25 for completion of the baseline visit, US $25 for completion of the randomization visit, US $50 for completion of the postintervention visit, and US $50 for device return.

### Recruitment

Participants were recruited from September 2022 to September 2023 through Vanderbilt University Medical Center outpatient clinics, the Vanderbilt University Medical Center employee distribution list, and ResearchMatch.org. Clinic patients received an opt-out letter or a message through the My Health at Vanderbilt app after electronic health record screening, followed by staff contact. Additionally, participants recruited via the Vanderbilt University Medical Center employee distribution list or ResearchMatch.org reached out to the study team after being notified by email about the study. Recruitment focused on participants residing near Nashville, Tennessee, due to blood sample collection.

### Study Visit Schedule

The study included 3 visits: baseline, randomization, and postintervention. At baseline, eligible participants provided written informed consent, completed surveys, and underwent cardiometabolic assessments, including anthropometrics, blood pressure, and dried blood spot collection. Participants then wore the activPAL3 (PAL Technologies) devices on the thigh and a continuous glucose monitoring (CGM) device (FreeStyle Libre Pro; Abbott) for 7 days, 24 hours per day, and completed a daily sleep diary and a device wear log during this period. After 7 days, participants returned the CGM and activPAL3 devices in person and were randomized into either the Sit Less program or the control group (randomization visit). Following the 12-week intervention or control period, participants completed the same assessments and wore both devices again for 7 days (postintervention visit). Devices were returned by mail, and all participants received their pre- and postintervention data after study completion.

### Randomization

Randomization occurred within each disease group. A 1:1 randomization list was created by a statistician and uploaded into the REDCap randomization module. Participants were informed of their group assignment immediately during their randomization visit. Participants and the interventionist were not blinded to the allocation due to the nature of the intervention program. However, outcome assessors and data analysts were blinded to the allocation.

### Sit Less Intervention

#### Overview

**Figure 1.**
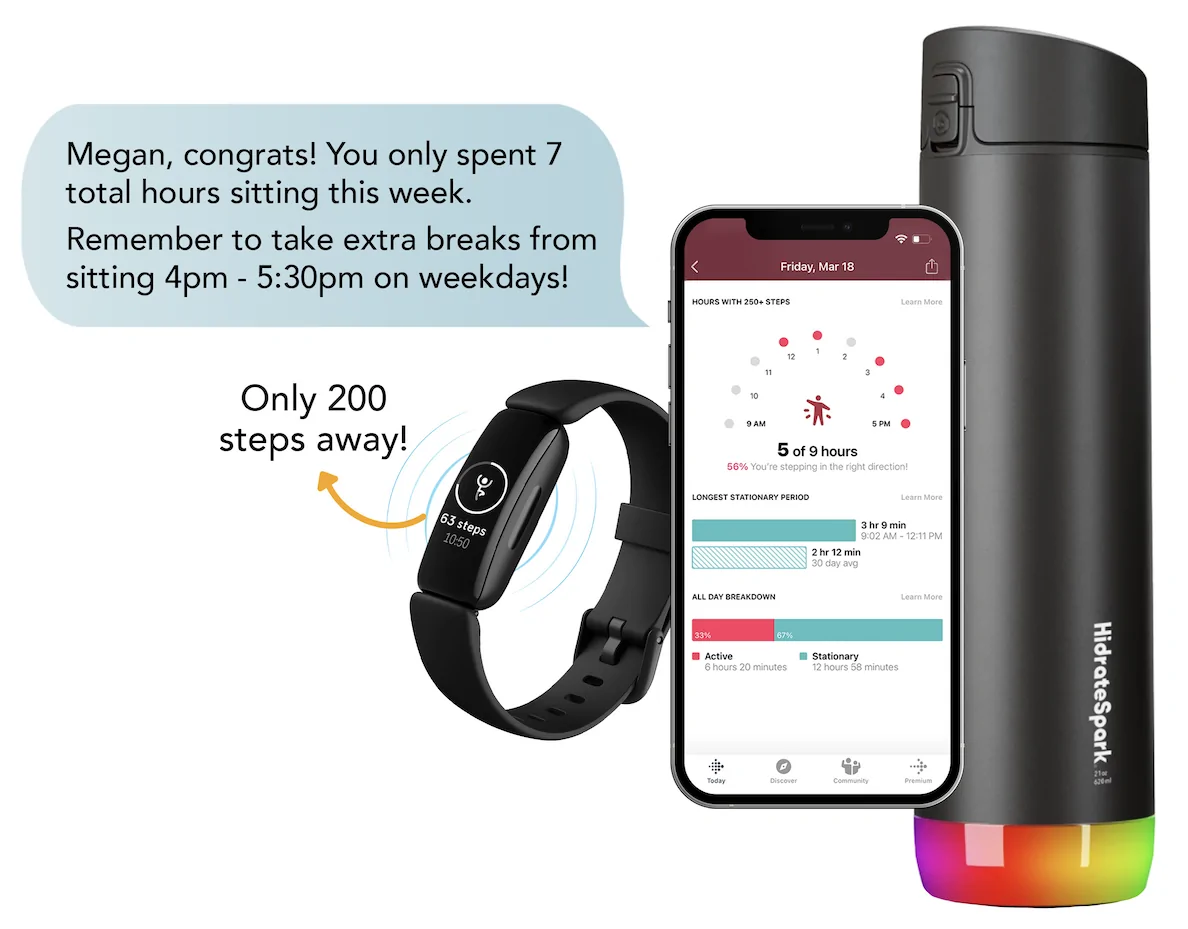
Overview of a multiple technology–based sedentary behavior reduction intervention (“Sit Less” program).

The 12-week Sit Less program was developed based on a Habit Formation framework [20] to target sedentary behavior. The Sit Less program included (1) one in-person instructional and goal-setting session at the randomization visit, (2) the use of a wearable device (Fitbit), (3) a smart water bottle (HidrateSpark), and (4) 3 weekly tailored text messages for behavior reinforcement and weekly goal monitoring. Figure 1 describes the Sit Less program. The overall goals of the Sit Less program were (1) to reduce daily sedentary time by 120 minutes, and (2) to achieve a sedentary break (standing or walking) for 5 minutes every 30 minutes by the end of the intervention period. Participants were asked to gradually reduce their sedentary time by at least 10 minutes, up to a maximum of 30 minutes, per week [21]. Detailed information regarding the intervention is described elsewhere [18].

#### Instructional or Goal-Setting Session

The principal investigator reviewed the activPAL data with Sit Less participants to identify targetable prolonged sedentary bouts. Using a modified Top Ten Tips (TTT) booklet—adapted from Habit Formation Theory—the principal investigator guided participants in developing habits to take frequent sedentary breaks. The original TTT booklet, which included stretching and muscle-strengthening activities [22], was revised to 8 tips focused specifically on reducing sedentary behavior [23]. Sit Less participants completed weekly worksheets to set goals and plan actions to reduce sedentary time based on the selected tips.

#### Fitbit

Fitbits (Inspire model) were used to support participants as they self-monitor sedentary hours (ie, “total stationary hours” and “longest stationary period”) and to provide real-time sedentary break prompts (ie, “Move alert”). The goal was to help participants become aware of their habitual sedentary behavior [24]. The Fitbit “Move alert” alerts participants after 60 minutes of sitting, prompting a 2‐ to 3-minute walk (≈250 steps) to reset the alert. Participants were advised to wear the Fitbit during waking hours, optionally at night, and to synchronize weekly. Participants who failed to synchronize or wear the Fitbit for 2 days received a reminder text; continued noncompliance for another 2 days prompted a call from research staff to troubleshoot issues [18].

#### HidrateSpark Smart Water Bottle

The smart water bottle (HidrateSpark Smart Water Bottle) was used to motivate participants to break their prolonged sitting and stand up and move by frequently visiting the restroom and refilling the bottle. The water bottle used flashing lights and phone notifications to remind participants to drink water if they did not meet personalized hydration targets based on their body weight, age, sex, and activity levels.

#### Weekly Text Messages

Participants received 3 algorithm-derived, Fitbit-based tailored messages: Thursday (midweek sedentary behavior summary), Sunday (encouragement for achieving the weekly goal), and Monday. The Monday message included feedback on prior week goals (based on Fitbit), a sedentary behavior summary, 1 TTT tip, and a prompt to set new goals for sitting time and the duration and frequency of sitting breaks. These messages supported habit formation and weekly planning to reduce sitting time and increase sitting breaks. The detailed algorithm and contents are described elsewhere [18].

### Control Group

The control group received standard care and the American Heart Association’s “Answers by Heart” booklet [25]. This booklet guides tracking and managing body weight, blood pressure, and cholesterol levels. The booklet also notes general advice on healthy cooking and physical activity [25]. The control group did not receive a Fitbit, a smart water bottle, or tailored text messages.

### Study Measures

#### Feasibility

Feasibility was evaluated by assessing recruitment or enrollment rates, dropout rates (percentage of participants who withdrew consent among enrolled participants), and adherence to the Sit Less program, defined as (1) ≥75% of participants using the Fitbit for ≥5 valid days per week for 75% of the study duration and (2) engagement with ≥80% of goal setting texts [26].

#### Acceptability

Acceptability of the Sit Less program was measured through satisfaction surveys by combining items from 2 different questionnaires [27,28]. The Lyons et al [27] questionnaire assessed overall program satisfaction, ease of use, and continued intention for each component and was adapted to evaluate Sit Less technological tools (Fitbit, app, and smart water bottle). The Burner et al [28] questionnaire focused on the text messaging aspect, examining content relevance, motivational degree, frequency, and timing of messages. Responses were rated on a 5-point Likert scale, with 75% agreement indicating acceptability.

#### Primary Outcome: Sedentary Behavior

Using the activPAL3 devices, we measured sedentary behavior as total daily sitting time, prolonged sitting time (time spent in sedentary bouts ≥30 minutes and time spent in sedentary bouts ≥60 minutes), and the number of sit-to-stand transitions for 7 days at baseline and at postintervention. The activPAL3, worn on the thigh, accurately detects sitting and standing postures and has been validated against direct observation [29]. ActivPAL3 data were used for analysis if the participant wore the device for more than 10 hours on a given day, at least 3 days [30]. Data for analysis were extracted from the device using PAL analysis (activPAL, version 8.11) with the validated CREA algorithm, which closely matches diary data in classifying wake time activities [31].

#### Secondary Outcomes

##### Physical Activity

Daily time spent standing and walking (stepping) was measured using the activPAL3 and processed through the CREA algorithm.

##### Cardiometabolic Markers

Cardiometabolic biomarkers included glycemic outcomes and additional cardiometabolic measures. Glycemic outcomes comprised HbA_1c_ and CGM metrics. Fasting finger-prick blood samples were collected onto dried blood spot cards (ZRT Laboratory) for HbA_1c_ analysis. CGM (Abbott FreeStyle Libre Pro) was conducted over 7 days to assess 24-hour average glucose, glucose management indicator, glycemic variability, and time in range (70‐180 mg/dL) [32].

Additional cardiometabolic measures included BMI, waist and hip circumferences, waist-to-hip ratio, blood pressure, insulin, hs-CRP, and lipids. Anthropometric and blood pressure measurements were obtained using standardized procedures, and dried blood spot samples were analyzed for insulin, lipids, and hs-CRP [33,34]. Dried blood spot testing has shown a strong correlation with conventional serum tests, making it a reliable and convenient tool for screening for cardiometabolic risk factors [33,34].

##### Psychological Factors

We measured participant confidence in reducing sedentary behavior using the 12 items from the Self-Efficacy Questionnaire for Physical Activity and Sedentary Behavior (previously reported Cronbach α=0.79 and 0.85) [35]. The items assess the level of confidence for specific sitting behaviors and sedentary breaks. Habit strength for sedentary behavior was assessed using the validated Self-Report Habit Index measure (previously reported Cronbach α=0.91, 0.82, and 0.83) [36]. This 7-item index was adapted to assess the degree to which sedentary breaks (standing or walking) became habitual [36]. Depressive symptoms were measured using the Patient Health Questionnaire-9 (PHQ-9) [37].

##### Demographic and Clinical Characteristics

Demographics, socioeconomic characteristics, medical history, current medication, tobacco use, and alcohol intake were collected via self-report. Participants were asked about their level of activity associated with their current occupation, and responses ranged from “mostly sedentary” (eg, desk-based job) to “very active and physical” (eg, manual labor). Using the 16-item Rapid Eating and Activity Assessment for Participants Short Version, we determined participants’ dietary habits [38].

### Statistical Analysis

Statistical analyses were conducted using SPSS Statistics 29 (IBM Corp) and STATA 18 (StataCorp). We summarized nominal and ordinal variables via frequency distributions. Continuous variables were summarized using means and SDs when normally distributed, such as the primary outcome of sedentary behavior and psychological factors, including self-efficacy for physical activity and sedentary behavior and habit strength for sedentary behavior. Medians with IQRs were also used when distributions were skewed or contained extreme outliers. This applied to continuous demographic and clinical characteristics, as well as secondary outcome variables, including physical activity, cardiometabolic and glucose monitoring biomarkers, and PHQ-9 scores. Baseline group differences were assessed using chi-square tests of independence (nominal and ordinal) and Mann-Whitney (continuous) tests.

We evaluated the intervention effects in the following steps. First, we calculated the change in each outcome variable from baseline to postintervention for each participant. Within-group changes were generated using median (for skewed variables) and mean (for normally distributed variables), with bootstrapped 95% CIs. Second, we conducted between-group analyses using generalized linear models with bias-corrected robust (Huber sandwich estimator, STATA) SEs to evaluate the effects of the intervention on primary and secondary outcomes. Each model included the corresponding baseline value of the outcome, cohort (CVD vs T2D), and the interaction between study group and cohort. Baseline outcome values were included to account for baseline variability, while the cohort and the cohort×group interaction term were included to account for known differences in cardiometabolic responses between the 2 clinical cohorts and to assess whether intervention effects differed by cohort. Leisure-time exercise was additionally included because it was the only baseline variable that differed significantly between groups and was considered clinically relevant to the outcomes. To maintain model parsimony and reduce the risk of overfitting, no additional covariates were included beyond those with clear clinical or methodological justification.

For each model, multicollinearity among the independent variables was assessed first. There was no multicollinearity problem among the variables for all models. We then conducted each model using the Gaussian distribution with the identity link function and evaluated the residuals for indications of extreme influence, lack of normality, and/or heteroscedasticity. When assumptions were violated, models were reestimated using a Gamma distribution with a log link, and the residuals were reevaluated. We also conducted the same analyses using transformed variables and the Gaussian distribution, with similar findings resulting from the models using the Gamma, log link function, enabling us to generate estimated mean differences on the original variable scale of measurement. A few biomarker variables had extreme outliers. Those variables could not be addressed through transformation or alternative model specifications. Given the small sample size, extreme values were winsorized by replacing them with the next highest observed value. Model assumptions were reevaluated following this adjustment. Final generalized linear models with appropriate distributions and link functions were used, and robust SEs were applied to estimate adjusted mean differences with 95% CIs. These estimates were used to evaluate the effect of the study group and to provide measures of effect size. All analyses were conducted using an intention-to-treat approach without data imputation.

## Results

### Participants

**Figure 2.**
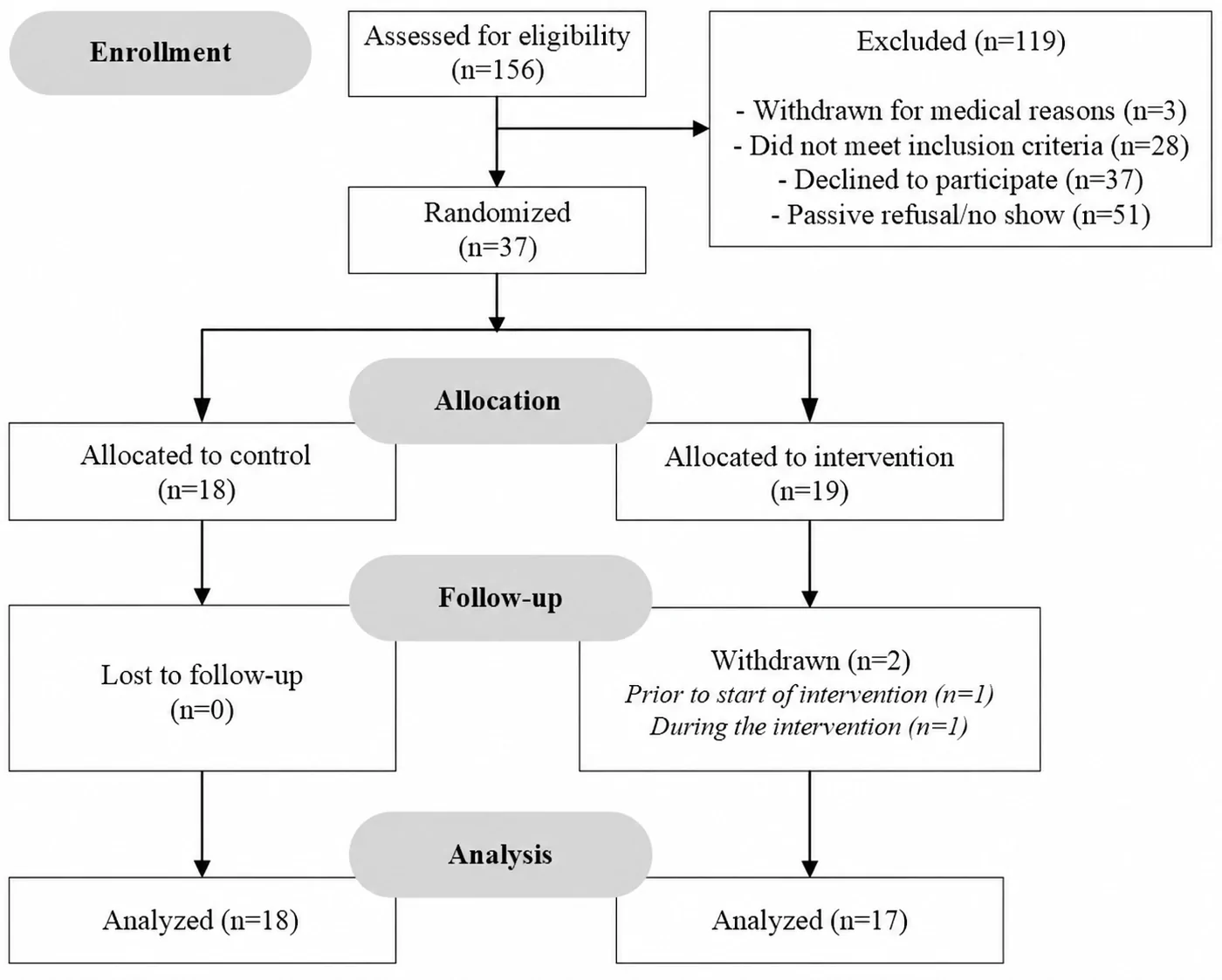
The CONSORT (Consolidated Standards of Reporting Trials) flow diagram for the Sit Less study with type 2 diabetes and cardiovascular disease cohort.

Of 156 participants who were contacted and assessed for eligibility, 128 (80%) met inclusion criteria, and of those, 37 (30%) completed all baseline assessments and were randomized (Figure 2). Of the 37 randomized participants, 19 (51%) were randomly assigned to the Sit Less group, and 18 (49%) were assigned to the control group. One (5%) participant from the Sit Less group completed baseline assessments but withdrew before the start of the intervention due to privacy concerns about the Fitbit displaying text messages and incoming calls. Another Sit Less group participant withdrew during the intervention due to a Fitbit data recording error. This participant used a walker, and because of the lack of wrist movement while walking with the walker, the Fitbit did not capture any steps or movement. Therefore, 35 (95%) of 37 participants completed the study and were included in the analysis (Sit Less: n=17; and control: n=18).

### Baseline Demographics and Clinical Characteristics

The median age of the final sample was 67 (IQR 54‐72) years. With the exception of leisure time exercise at baseline, no significant differences between groups were found in the demographic or clinical characteristics (Table 1). At baseline, 7 (39%) of 18 participants in the control group and 14 (82%) of 17 participants in the Sit Less group reported leisure-time exercise (*P*=.009).

Both the Sit Less and control groups wore the activPAL continuously (24 h/d), with minimal nonwear time (median 0 min/d, IQR 0‐0). Participants provided a median of 7 valid wear days at both preintervention (IQR 7‐8) and postintervention (IQR 6‐7). On average, the participants spent a median of 624 minutes sitting (IQR 521‐686), 182 minutes standing (IQR 108‐230), 72 minutes stepping (IQR 45‐86), 32 minutes in secondary lying positions (IQR 0‐83), and 490 minutes sleeping (IQR 450‐523). No significant between-group differences were found in nonwear time and baseline activities except for stepping time.

**Table 1.** Baseline demographic and clinical characteristics.

Sample characteristics	Overall (N=35)	Control (n=18)	Sit Less (n=17)	*P* value^a^
Age (y), median (IQR)	67.0 (54-72)	64.5 (54-73)	68.0 (55-72)	.78
Dietary habits (REAP-S^b^), median (IQR)	28.0 (26-32)	30.0 (25-32)	28.0 (26-31)	.79
Condition cohort, n (%)				.36
Diabetes	13 (37.1)	8 (44.4)	5 (29.4)	
CVD^c^	22 (62.9)	10 (55.6)	12 (70.6)	
Gender, n (%)				.72
Female	13 (38.2)	7 (41.2)	6 (35.3)	
Male	21 (61.8)	10 (58.8)	11 (64.7)	
Married or partnered, n (%)				.72
No	13 (38.3)	6 (35.3)	7 (41.2)	
Yes	21 (61.8)	11 (64.7)	10 (58.8)	
Race or ethnicity, n (%)				.99
Non-Hispanic White	28 (82.4)	14 (82.4)	14 (82.4)	
Non-Hispanic Black	4 (11.8)	2 (11.8)	2 (11.8)	
Asian, Hispanic, or other	2 (5.9)	1 (5.9)	1 (5.9)	
Education level, n (%)				.27
Less than college	11 (32.4)	7 (41.2)	4 (23.5)	
College or higher	23 (67.6)	10 (58.8)	13 (76.5)	
Income sufficiency, n (%)				.55
Poorly or not well	3 (8.8)	2 (11.8)	1 (5.9)	
Well or very well	31 (91.2)	15 (88.2)	16 (94.1)	
Employment, n (%)				.44
Full	14 (41.2)	8 (47.1)	6 (35.3)	
Part	7 (20.6)	2 (11.8)	5 (29.4)	
Unemployed, retired, or other	13 (38.2)	7 (41.2)	6 (35.3)	
If employed, the job (overall, n=21; control, n=10; Sit Less, n=11)				.70
Active	5 (23.8)	2 (20.0)	3 (27.3)	
Sedentary	16 (76.2)	8 (80.0)	8 (72.7)	
Alcohol intake (last 12 mo), n (%)				.48
No	8 (22.9)	5 (27.8)	3 (17.6)	
Yes	27 (77.1)	13 (72.2)	14 (82.4)	
Smoke cigs (last 12 mo), n (%)				.26
No	31 (88.6)	17 (94.4)	14 (82.4)	
Yes	4 (11.4)	1 (5.6)	3 (17.6)	
Leisure time exercise, n (%)				.009
No	14 (40.0)	11 (61.1)	3 (17.6)	
Yes	21 (60.0)	7 (38.9)	14 (82.4)	
Health conditions, n (%)				
High blood pressure	19 (54.3)	11 (61.1)	8 (47.1)	.40
High cholesterol	21 (61.8)	11 (64.7)	10 (58.8)	.72
Arthritis	23 (65.7)	13 (72.2)	10 (58.8)	.40
Sleep apnea	15 (42.9)	8 (44.4)	7 (41.2)	.85
Other sleep problems	14 (40.0)	7 (38.9)	7 (41.2)	.89

a *P* values were calculated using the Mann-Whitney *U* test for continuous variables with skewed distributions (age and REAP-S score) and the chi-square test for categorical variables.

b REAP-S: Rapid Eating and Activity Assessment for Participants Short Version.

c CVD: cardiovascular disease.

### Feasibility

**Figure 3.**
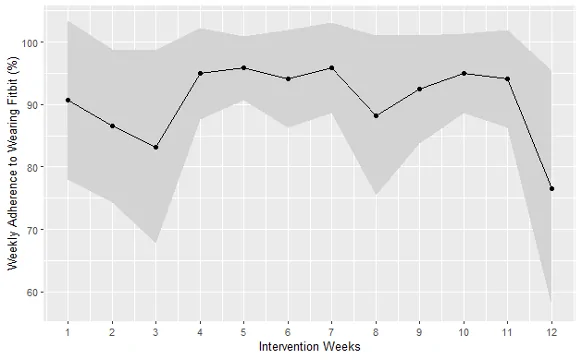
Weekly average adherence to wearing a Fitbit over 12 weeks. The gray area indicates the 95% CI.

As noted earlier, among the 37 participants enrolled, 35 (95%) completed all study activities. The Sit Less group had a dropout rate of 2 (11%) of 19; the reason for the dropout was privacy concerns about the Fitbit displaying text messages and incoming calls and Fitbit’s data recording errors. Adherence to the Sit Less program was high according to our adherence definition (Figure 3). Fifteen (88%) of 17 participants wore the Fitbit for more than 75% of the intervention period. On average, participants wore their Fitbit for 76 of 84 days. Weekly, the Sit Less participants had a median of 6.8 valid wear days per week—where a valid day indicated wearing the Fitbit for more than 10 hours. Daily median Fitbit wear time was 928 minutes per day (IQR 902‐944). Adherence to Fitbit wear peaked at weeks 5 and 7, with 96% wearing the device, and was maintained throughout the study period, except for week 12, when adherence dropped to 76%.

**Figure 4.**
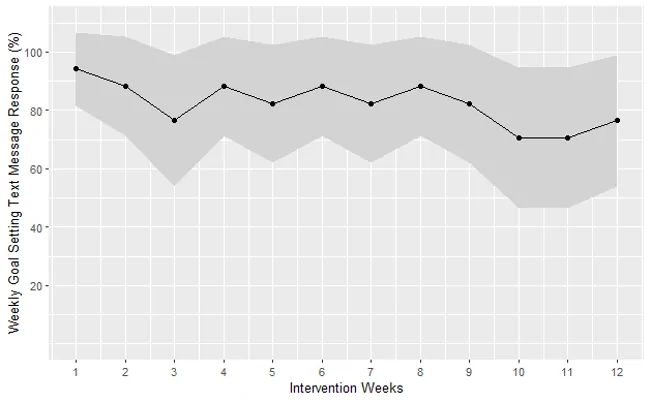
Weekly average adherence to responding to goal-setting text messages over 12 weeks. The gray area indicates the 95% CI.

For text message responses, participants were diligent in setting weekly goals and replying to us (Figure 4). The average rate of text message response over 12 weeks was 81%. Until week 9, participants consistently responded to goal-setting messages, with adherence rates ranging from 91% to 83%. However, in the last 3 weeks of the program, response rates decreased, ranging between 58% and 66%.

### Acceptability

A total of 17 Sit Less participants completed the satisfaction survey. Fifteen (88%) of 17 participants indicated that they would recommend the program to family and friends. Fifteen (88%) of 17 participants indicated that the program increased awareness of their sedentary behavior. Sixteen (94%) of 17 participants liked wearing the Fitbit monitor; 13 (77%) of 17 participants would continue checking sitting hours on Fitbit, and 11 (65%) of 17 participants indicated that the “Move Alert” from Fitbit was useful. The majority (11/17, 65%) of participants also indicated that they found the smart water bottle convenient to use. Fifteen (88%) of 17 Sit Less participants either agreed (47%) or remained neutral (41%) about whether they would like to receive text messages for a longer period. The majority (13/17, 77%) of participants either agreed or were neutral that the tips and advice felt specific to them, and they were motivated by the text messages. Most participants (16/17, 94%) indicated that the number of text messages was “just right.”

### Primary Outcome: Sedentary Behavior

Group differences in sedentary behavior at baseline and postintervention are presented in Table 2. Overall, the Sit Less intervention showed consistent directional improvements compared with the control group, although between-group differences were not statistically significant. For total sitting time, the control group increased from 588.3 (SD 100.2) to 593.6 (SD 83.4) minutes per day, whereas the Sit Less group decreased from 643.6 (SD 113.0) to 605.0 (SD 111.1) minutes per day. After adjustment for baseline values, cohort, cohort×group interaction, and leisure-time exercise, the Sit Less group showed a nonsignificant reduction of 17.3 minutes per day in total sitting time compared with the control group at postintervention (95% CI −63 to 28; *P*=.45).

**Table 2.** Changes in sedentary behavior outcomes from baseline to postintervention by study group (N=35; control: n=18; Sit Less: n=17; all baseline between-group differences were *P*>.05).

Outcomes	Baseline, mean (SD)	Postintervention, mean (SD)	Mean change (95% CI)	Estimated adjusted mean difference (95% CI)^a^	*P* value^b^
Sitting time (min/d)				−17.3 (−63 to 28)	.45
Control	588.3 (100.2)	593.6 (83.4)	5.3 (−38 to 50)		
Sit Less	643.6 (113.0)	605.0 (111.1)	−38.6 (−71 to 4)		
Sitting and secondary lying time (min/d)				−21.7 (−76 to 32)	.43
Control	660.2 (112.0)	669.4 (83.5)	9.2 (−47 to 70)		
Sit Less	679.4 (129.4)	642.0 (113.5)	−37.4 (−80 to 11)		
Time spent sitting bouts >30 min (min/d)				−39.5 (−100 to 21)	.20
Control	345.0 (144.0)	355.6 (106.4)	10.7 (−41 to 65)		
Sit Less	355.5 (135.7)	304.8 (140.5)	−50.7 (−115 to 8)		
Time spent sitting bouts >60 min (min/d)				−49.9 (−102 to 2)	.06
Control	203.1 (144.8)	205.0 (116.6)	1.9 (−47 to 44)		
Sit Less	194.4 (113.0)	142.4 (112.8)	−52.0 (−111 to 4)		
Sit-to-stand transitions (n per day)				4.2 (−2 to 10)	.13
Control	36.2 (13.7)	36.8 (12.0)	0.6 (−2 to 3)		
Sit Less	43.7 (9.9)	46.6 (8.9)	2.9 (−3 to 9)		

a Test of the difference between adjusted marginal means using a generalized linear model with a Gaussian distribution and identity link, with robust SEs (*χ*² statistic, df=1).

b Adjusted for study cohort, cohort×group interaction, baseline value, and leisure-time exercise; estimates generated using robust SEs.

Time spent in sedentary bouts of >30 minutes increased in the control group and decreased in the Sit Less group (Table 2), with a nonsignificant adjusted between-group difference of −39.5 minutes per day (95% CI −100 to 21; *P*=.20). For sedentary bouts of >60 minutes, the control group showed minimal change (1.9 min/d; 95% CI −47 to 44), whereas the Sit Less group decreased by 52.0 minutes per day (95% CI −111 to 4), with an adjusted difference of −49.9 minutes per day (95% CI −102 to 2; *P*=.06).

Sit-to-stand transitions were unchanged in the control group (36.2, SD 13.7 vs 36.8, SD 12.0) but increased in the Sit Less group (43.7, SD 9.9 vs 46.6, SD 8.9), with a nonsignificant adjusted mean difference of 4.2 transitions per day (95% CI −2 to 10; *P*=.13).

### Secondary Outcome: Physical Activity

Group differences in physical activity at baseline and postintervention are presented in Table 3. Although not statistically significant, participants in the Sit Less group increased both standing and stepping time by approximately 10 minutes more than those in the control group, despite variability in change scores. For standing time, the control group decreased from 170.5 (IQR 99‐255) to 158.1 (IQR 115‐256) minutes per day, whereas the Sit Less group increased from 182.9 (IQR 144‐228) to 193.4 (IQR 144‐239). The adjusted between-group difference was 9.7 minutes per day (95% CI −52 to 71; *P*=.76), but it was not significant.

For stepping time, baseline values differed between groups (control: median 49.7 min/d, IQR 37‐75; Sit Less: median 80.8 min/d, IQR 52‐108; *P*=.01). Following the intervention, the control group and the Sit Less group both increased stepping time, but there was no statistically significant between-group difference following the intervention (adjusted mean difference 9.6 min/d, 95% CI −15 to 34; *P*=.44).

Step counts also increased in both groups; however, there was no statistically significant between-group difference following the intervention (adjusted mean difference 711.4 steps per day, 95% CI −983 to 2406; *P*=.41).

**Table 3.** Changes in physical activity outcomes from baseline to postintervention by study group (N=35; control: n=18; Sit Less: n=17)^a^.

Outcomes	Baseline, median (IQR)	Postintervention, median (IQR)	Median change (95% CI)	Estimated adjusted mean difference^b^ (95% CI)	*P* value^c^
Standing time (min/d)				9.7 (−52 to 71)	.76
Control	170.5. (99‐255)	158.1 (115‐256)	26.1 (−39 to 59)		
Sit Less	182.9 (144‐228)	193.4 (144‐239)	−0.5 (−30 to 44)		
Stepping time (min/d)				9.6 (−15 to 34)	.44
Control	49.7 (37-75)^d^	58.8 (45‐80)	11.7 (−1 to 19)		
Sit Less	80.8 (52‐108)	89.5 (53‐107)	−0.8 (−11 to 22)		
Step counts (steps per day)				711.4 (−983 to 2406)	.41
Control	3682 (2680‐5248)	4085 (3518‐6274)	991 (−145 to 1340)		
Sit Less	5759 (4254‐8337)	6299 (4216‐8594)	36 (−447 to 1427)		

a Due to moderate skewness, median (IQR) and median change used to describe the observed distributions.

b Adjusted for the main effect of study cohort, the interaction of study cohort with study group, exercise during leisure time prior to study, and baseline values; estimated mean differences generated using robust SEs.

c Test of the difference between adjusted marginal means using a generalized linear model with a Gaussian distribution and identity link, with robust standard errors (*χ*² statistic, df=1).

d Baseline between-group difference, Mann-Whitney *U* test, *P*<.05. All other baseline between-group differences were *P*>.05.

### Secondary Outcomes: Cardiometabolic Markers

Glycemic outcomes at baseline and postintervention are summarized in Table 4. HbA_1c_ levels were significantly lower in the Sit Less group compared with the control group at postintervention (adjusted mean difference −0.4%, 95% CI −0.8 to −0.1; *P*=.04), with a decrease observed in the Sit Less group (median change −0.4%, 95% CI −0.6 to −0.2) and minimal change in the control group (median change 0.1%, 95% CI −0.1 to 0.5). Although not statistically significant, CGM outcomes were directionally consistent with HbA_1c_. Adjusted mean differences indicated lower glucose management index (−0.1%, 95% CI −0.4 to 0.1; *P*=.16) and average glucose level (−6.0 mg/dL, 95% CI −14.9 to 2.8; *P*=.18) in the Sit Less group compared with the control group at postintervention (Table 4). Participants wore the CGM device for a median of 9 days in both groups, with no between-group differences. Sensitivity analyses excluding extreme outliers were conducted for HbA_1c_ (n=1 control), CGM-based glucose management index (n=2 controls), and average glucose level (same 2 controls as GMI). While the estimated mean differences and *P* values changed slightly, interpretations of the results remained the same. Those summaries and findings are provided in Multimedia Appendix 1.

**Table 4.** Changes in glycemic outcomes from baseline to postintervention by study group (N=35)^a^.

Outcomes	Baseline, median (IQR)	Postintervention, median (IQR)	Median change (95% CI)	Estimated adjusted mean difference^b^ (95% CI)	*P* value
HbA_1c_^c^, % (control: n=18 and Sit Less: n=16)				−0.4 (−0.8 to −0.1)	.04^d^
Control	5.5 (4.3‐5.7)	5.3 (4.8‐5.9)	0.1 (−0.1 to 0.5)		
Sit Less	5.0 (4.7‐5.9)	4.9 (4.4‐5.5)	−0.4 (−0.6 to −0.2)^e^		
Glucose management index, % (control: n=15 and Sit Less: n=15)				−0.1 (−0.4 to 0.1)	.16^f^
Control	5.7 (5.6‐6.6)	6.0 (5.4‐6.5)	−0.1 (−0.5 to 0.1)		
Sit Less	6.0 (5.5‐6.4)	5.7 (5.5‐6.2)	−0.1 (−0.2 to 0.1)		
Average glucose level, mg/dL (control: n=15 and Sit Less: n=15)				−6.0 (−14.9 to 2.8)	.18^f^
Control	103.0 (96‐136)	113.0 (90‐133)	−2.0 (−18 to 5)		
Sit Less	113.0 (94‐127)	100.0 (93‐120)	−3.0 (−8 to 1)		
Glycemic variability (coefficient of variation), % (control: n=15 and Sit Less: n=15)				0.6 (−3.5 to 4.6)	.78^g^
Control	21.7 (18‐32)	22.2 (19‐30)	−0.1 (−2.8 to 1.8)		
Sit Less	20.3 (16‐34)	20.9 (17‐27)	1.3 (0.3 to 2.0)^e^		
Time in range (70‐180 mg/dL), % (control: n=15 and Sit Less: n=15)				2.1 (−4.8 to 9.1)	.54^g^
Control	91.0 (75‐94)	94.0 (71‐96)	0.0 (−5 to 5)		
Sit Less	94.0 (85‐98)	94.0 (86‐98)	1.0 (0 to 1)		

a Due to extreme skewness, median (IQR) and median change used to describe the observed distributions; all baseline between-group differences were *P*>.05.

b Adjusted for study cohort, cohort×group interaction, baseline value, and leisure-time exercise; estimates generated using robust SEs.

c HbA_1c:_ hemoglobin_ A1c_.

d Test of the difference between adjusted marginal means using a generalized linear model with a Gamma distribution and log link function, with robust SEs (chi-square statistic, df=1). One extreme outlier in the control group at postintervention was winsorized prior to analysis.

e Within-group change statistically significant based on bootstrapped 95% CI.

f Test of the difference between adjusted marginal means using a generalized linear model with a Gamma distribution and log link function, with robust SEs (chi-square statistic, df=1). Two extreme outliers in the control group, one at baseline and another at postintervention were winsorized prior to analysis.

g Test of the difference between adjusted marginal means using a generalized linear model with a Gaussian distribution and identity link, with robust SEs (chi-square statistic, df=1).

No statistically significant between-group differences were observed for other cardiometabolic outcomes, including weight, BMI, waist circumference, insulin, or blood pressure. Total cholesterol and low-density lipoprotein decreased and high-density lipoprotein increased, whereas triglycerides increased; however, none of these adjusted between-group differences were statistically significant (Multimedia Appendix 2).

### Secondary Outcomes: Psychological Outcomes

Psychological outcomes at baseline and postintervention are summarized in Table 5. No adjusted between-group differences were observed for sedentary behavior habit strength (adjusted mean difference 0.0; 95% CI −0.7 to 0.8; *P*=.89). Self-efficacy for increasing physical activity increased in the control group but decreased in the Sit Less group, with a significant between-group difference observed for moderate activity (adjusted mean difference −0.6, 95% CI −1.1 to −0.2; *P*=.003; Table 5). For depressive symptoms (PHQ-9), the adjusted between-group difference at postintervention was not statistically significant.

**Table 5.** Changes in psychological outcomes from baseline to postintervention by study group (N=35)^a^.

Outcomes	Baseline, mean (SD)	Postintervention, mean (SD)	Mean change (95% CI)	Estimated adjusted mean difference^b^ (95% CI)	*P* value^c^
Sedentary behavior habit strength (minimum=1 and maximum=7)				0.0 (−0.7 to 0.8)	.89
Control	6.0 (0.7)	5.8 (0.9)	−0.2 (−0.6 to 0.3)		
Sit Less	5.9 (1.1)	5.8 (1.0)	−0.1 (−0.7 to 0.4)		
Self-efficacy in reducing sitting (minimum=1 and maximum=5)				−0.4 (−0.9 to 0.2)	.15
Control	3.6 (0.9)	3.3 (0.9)	−0.3 (−0.8 to 0.2)		
Sit Less	3.5 (0.6)	3.1 (1.0)	−0.4 (−0.8 to 0.1)		
Self-efficacy in increasing light physical activity (minimum=1 and maximum=5)				−0.4 (−0.9 to 0.1)	.07
Control	3.6 (0.9)	3.6 (0.7)	0.1 (−0.3 to 0.5)		
Sit Less	3.6 (0.6)	3.5 (0.8)	−0.1 (−0.5 to 0.2)		
Self-efficacy in increasing moderate physical activity (minimum=1 and maximum=5)				−0.6 (−1.1 to −0.2)	.003
Control	3.3 (1.2)	3.7 (1.2)	0.4 (0.0 to 0.9)		
Sit Less	3.8 (1.2)	3.8 (1.1)	−0.1 (−0.5 to 0.3)		
PHQ-9^d^ (minimum=0 and maximum=27), median (IQR)				−0.2 (−1.4 to 1.1)	.76
Control	4.0 (1-10)	3.0 (1-7)	−1.0 (−2 to 0)		
Sit Less	4.0 (2-5)	2.0 (1-5)	−1.0 (−2 to 0)		

a Control: n=18; Sit Less: n=17; all baseline between-group differences were *P*>.05.

b Adjusted for the main effect of study cohort, the interaction of study cohort with study group, exercise during leisure time prior to study, and baseline values; estimated mean differences generated using with robust SEs.

c Test of the difference between adjusted marginal means using a generalized linear model with a Gaussian distribution and identity link, with robust SEs (chi-square statistic, df=1).

d Patient Health Questionnaire-9 (PHQ-9) scores are reported as median (IQR) at baseline, postintervention, and for median change due to a skewed distribution. All other psychological values are presented as mean (SD) unless otherwise specified.

## Discussion

### Principal Findings

Frequent, short breaks (walking 5 min for every hour sitting) are practical, low-burden alternatives to exercise, proven to improve glucose and insulin levels [7,39]. However, intervention studies on sedentary breaks in T2D or CVD are limited and mostly short-term laboratory studies [7,39]. This is the first study integrating Fitbit devices and smart water bottle technologies and algorithm-driven text messages to encourage frequent sedentary breaks. Focusing on sedentary breaks addresses barriers associated with traditional exercise programs—such as fear of hypoglycemia, cardiac discomfort, and time constraints [1,6,11]—while concurrently reducing overall sedentary time and increasing standing and walking duration. The intervention demonstrated high feasibility, acceptability, and adherence within the patient with cardiometabolic diseases groups over a 12-week period, characterized by high engagement with text messaging and consistent use of Fitbits. Participants in the Sit Less group also reported that wearable technology and smart water bottles were instrumental in breaking up their prolonged sitting. Notably, these positive outcomes were observed in a participant group with a median age of 67 years, indicating the potential of our multitechnology-based Sit Less program to reduce sedentary time and foster more active lifestyles among older adults with cardiometabolic conditions.

In this randomized controlled pilot trial, we were underpowered to detect intervention effects on outcomes, and CIs are wide; however, we still detected some directional changes in outcomes of interest in the Sit Less group relative to the control group. The Sit Less program was associated with reductions in total daily sedentary time compared with the control group, although between-group differences were not statistically significant. Over 12 weeks, the control group increased sitting time by 5.3 minutes per day, whereas the Sit Less group reduced sitting time by 38.6 minutes per day, corresponding to an adjusted difference of −17.3 minutes per day. Considering that reducing and replacing 30 minutes of sedentary time yields significant cardiometabolic benefits and reduces the risk of developing CVD and mortality [21], the observed reductions in the Sit Less group may have clinical relevance for individuals with diabetes and/or CVD.

Consistent patterns were observed for prolonged sedentary behavior, with decreases in time spent in bouts longer than 30 and 60 minutes in the Sit Less group compared with the control group. Prolonged, uninterrupted sitting is more strongly associated with adverse cardiometabolic outcomes than interrupted sitting [40,41]. A recent meta-analysis, while accounting for total sedentary time and moderate-to-vigorous physical activity time, revealed that even a single break per hour can modestly improve BMI, waist circumference, inflammation, and blood pressure [12,13]. Experimental studies confirm that sedentary breaks can improve glucose levels by 2% to 17% and fasting insulin levels by 15% [12,14]. In line with this, Sit Less participants had more sedentary breaks, shorter prolonged sitting, and lower HbA_1c_ (via dried blood spot) than controls postintervention. Our findings suggest that the intervention was feasible for targeting prolonged sedentary time and provide preliminary suggestions for further investigation in adequately powered trials. However, given the pilot nature of the study, small sample size, and multiple outcomes examined, the HbA_1c_ finding should be interpreted cautiously. Future studies powered to detect intervention effects and mechanisms of effects are needed.

With regard to physical activity outcomes, the Sit Less group spent 10 minutes more on stepping activities and standing compared to the control group postintervention, after adjustments. This level of change may have been insufficient to improve some cardiometabolic outcomes, including blood pressure and anthropometric measures. Considering that replacing sedentary time with higher-intensity physical activity leads to more substantial improvements in cardiometabolic markers, future interventions should encourage not only standing but also engaging in higher-intensity stepping for greater cardiometabolic benefits.

Despite these improvements in objective outcomes, psychological outcomes (eg, self-efficacy for reducing sedentary behavior and habit strength) did not change. Moreover, self-efficacy for increasing moderate physical activity was significantly higher in the control group compared to the Sit Less group at postintervention after adjustment. One possible explanation is that participants in the Sit Less group may have become more aware of the challenges of replacing sedentary behavior with moderate levels of physical activity, a phenomenon known as response shift, which is commonly observed in self-reported outcomes in behavioral interventions [42]. However, it is also possible that the measures used were not sufficiently sensitive to detect short-term changes in psychological constructs such as habit formation or that the pilot sample size was underpowered to detect modest psychological effects. Future research could incorporate approaches such as postintervention pretests, in which participants retrospectively assess their baseline perceptions, to better capture these changes. Future research could use a postintervention pretest in which participants report after the intervention what they would have reported at pretest, given what they have learned through the intervention.

### Limitations

The goals of this pilot study were to assess the acceptability of the intervention and the feasibility of the study design. The small sample size aligns with these goals but limits our ability to make conclusions about the efficacy of the Sit Less intervention. The study was not powered to formally test hypotheses regarding intervention efficacy, and CIs were wide. Those findings should therefore be interpreted as a preliminary suggestion for testing the Sit Less intervention in a future fully powered trial. To maintain model parsimony and reduce the risk of overfitting, no additional exploratory covariates were included in the final models beyond those considered clinically or methodologically essential. Accordingly, the significant finding for HbA_1c_ should be interpreted with caution in light of the limited sample size and potential model instability.

In addition, several features of this study limit our generalizability. For instance, participants were recruited from a single hospital in Nashville, USA, and were predominantly White and highly educated. Although feasibility and acceptability were high in this sample, challenges such as varying comfort with technology and device usability may be more pronounced in other populations.

Moreover, baseline HbA_1c_ levels (median, approximately 5.0%‐5.5%) were within the nondiabetic or well-controlled range for many participants, and no participants had baseline HbA_1c_ levels >7.0%, further limiting generalizability. In addition, given the very small sample size within each cohort, the study is not designed to estimate differential treatment effects between CVD and T2D.

Finally, we are unable to determine the mechanisms driving change in objective outcomes. For instance, we did not collect objective data on smart water bottle use (eg, refill frequency), limiting our ability to assess its independent contribution to behavioral changes. Future larger studies, including those using multiple intervention conditions or fractional factorial designs that vary components, could examine how different components impact outcomes to understand mechanisms of change and seek to evaluate Sit Less in more diverse and clinically complex populations.

### Clinical Implications

Breaking up prolonged sitting should be a priority in the clinical management of patients with cardiometabolic diseases, as even short, frequent activity breaks can improve glycemic control and reduce sedentary time. The Sit Less program achieved these outcomes by integrating simple standing and walking breaks into daily routines, effectively overcoming common exercise barriers such as fear of hypoglycemia, cardiac discomfort, and time constraints. Its multitechnology was well received by older adults, reinforcing its suitability for patients with cardiometabolic diseases. In addition, the dropout of 1 participant due to inaccurate Fitbit data associated with walker use highlights a limitation of wrist-worn activity monitors in this population. Such devices may not accurately capture activity in individuals who rely on assistive devices, as arm movement is restricted. This should be considered in future studies and implementation efforts involving older or mobility-impaired cardiometabolic populations, where alternative monitoring approaches such as hip monitoring may be more appropriate.

### Conclusions

In conclusion, the Sit Less intervention effectively integrated multiple technologies to reduce sedentary behavior in patients with cardiometabolic diseases. By targeting prolonged sedentary time, the program has shown a trend to decrease in total sitting time, increase in sedentary breaks, and possible improvements in glycemic outcomes. Our approach, which minimizes typical exercise barriers and promotes short active breaks, has demonstrated high feasibility and acceptance, suggesting its promise for future large-scale interventions. While further research is necessary to confirm these findings and assess their impact on a range of cardiometabolic markers, the initial results advocate for health care strategies that promote sedentary breaks to manage cardiometabolic disease.

## Supplementary material

10.2196/56497 Multimedia Appendix 1 Sensitivity analyses of changes in glycemic outcomes from baseline to postintervention by study group.

10.2196/56497 Multimedia Appendix 2 Changes in additional cardiometabolic outcomes from baseline to postintervention by study group.

10.2196/56497 Checklist 1 CONSORT-EHEALTH (version 1.6.1)—Submission_Publication Form.

## Abbreviations

CGM: contiguous glucose monitor
CONSORT: Consolidated Standards of Reporting Trials
CVD: cardiovascular disease
HbA_1c_: hemoglobin A_1c_
Hs-CRP: high-sensitivity C-reactive protein
PHQ-9: Patient Health Questionnaire-9
T2D: type 2 diabetes
TTT: Ten Top Tips
