# Supplementary material for: Feasibility and Preliminary Efficacy of a “Sit Less” Program Leveraging Fitbit Tracking and Tailored Text Messages in Cardiometabolic Disease: Findings From 2 Parallel Randomized Controlled Trials in Coronary Artery Disease and Type 2 Diabetes
Source: JMIR Mhealth Uhealth. 2026 Aug 25;14:e56497. doi: 10.2196/56497 (PMC13505886; doi:10.2196/56497)
Supplement: Supplementary file 2 [file mhealth-v14-e56497-s001.docx]

**Multimedia Appendix 1. Sensitivity Analyses of Changes in Glycemic Outcomes from Baseline to Post-Intervention by Study Group**

|  | Baseline  Median (IQR) | Post-Intervention  Median (IQR) | Median Change  (95% CI) | Estimated  Adjusted Mean Difference^a^  (95% C.I.) | *P* value |
| --- | --- | --- | --- | --- | --- |
| **HbA1c, %** (Control: n=17, Sit Less: n=16) | | | | | |
| Control | 5.4 (4.3−5.7) | 5.2 (4.7−5.7) | 0.0 (-0.1, 0.2) | -0.3 (-0.7, -0.1) | .04^b^ |
| Sit Less | 5.0 (4.7−5.9) | 4.9 (4.4−5.5) | -0.4 (-0.6, -0.2)^c^ |  |  |
| **Glucose management index, % (**Control: n=13, Sit Less: n=15) | | | | | |
| Control | 5.8 (5.4, 6.3) | 5.8 (5.4, 6.2) | 0.0 (-0.5, 0.1) | -0.1 (-0.3, 0.2) | .52^d^ |
| Sit Less | 6.0 (5.5, 6.4) | 5.7 (5.5, 6.2) | -0.1 (-0.2, 0.1) |  |  |
| **Average glucose level, mg/dL (**Control: n=13, Sit Less: n=15) | | | | | |
| Control | 103.0 (94, 123) | 104.0 (89, 118) | -2.0 (-18, 5) | -2.8 (-11.2, 5.8) | .52^d^ |
| Sit Less | 113.0 (94, 127) | 100.0 (93, 120) | -3.0 (-8, 1) |  |  |

Due to extreme skewness, median (IQR) and median change used to describe the observed distributions; all baseline between-group differences were P>.05.

^a^ Adjusted for study cohort, cohort × group interaction, baseline value, and leisure-time exercise; estimates generated using robust standard errors.

^b^Test of the difference between adjusted marginal means using a generalized linear model with a Gamma distribution and log link function, with robust standard errors (chi-square statistic, df=1). One extremely high outlier in the control group at post-intervention was removed.

^c^Within-group change statistically significant based on bootstrapped 95% CI.

^d^Test of the difference between adjusted marginal means using a generalized linear model with a Gamma distribution and log link function, with robust standard errors (chi-square statistic, df=1).

In the control group, one extremely high outlier in the baseline distribution and another in the post-intervention distribution were removed.
