# Supplementary material for: Feasibility and Preliminary Efficacy of a “Sit Less” Program Leveraging Fitbit Tracking and Tailored Text Messages in Cardiometabolic Disease: Findings From 2 Parallel Randomized Controlled Trials in Coronary Artery Disease and Type 2 Diabetes
Source: JMIR Mhealth Uhealth. 2026 Aug 25;14:e56497. doi: 10.2196/56497 (PMC13505886; doi:10.2196/56497)
Supplement: Supplementary file 3 [file mhealth-v14-e56497-s002.docx]

**Multimedia Appendix 2. Changes in additional cardiometabolic outcomes from baseline to post-intervention by study group**

|  | **Baseline,**  **Median (IQR)** | **Post-Intervention,**  **Median (IQR)** | **Median Change**  **(95% CI)** | **Estimated**  **Adjusted Mean Difference^a^**  **(95% C.I.)** | ***P* value** |
| --- | --- | --- | --- | --- | --- |
| **Weight, kg (Control: n=18, Sit Less: n=17)** | | | | | |
| Control | 102.2 (82,118) | 97.6 (84, 117) | -0.3 (-1.9, 1.2) | -0.2 (-2.0, 1.7) | .85^b^ |
| Sit Less | 91.6 (78, 104) | 88.0 (78, 102) | 0.3 (-0.7, 1.0) |  |  |
| **BMI, kg/m (Control: n=18, Sit Less: n=17)** | | | | | |
| Control | 31.8 (29, 38) | 31.6 (29, 38) | 0.0 (-0.3, 0.3) | 0.2 (-0.1, 0.6) | .18^b^ |
| Sit Less | 29.5 (26, 38) | 29.6 (27, 38) | 0.0 (-0.2, 0.3) |  |  |
| **Waist Circumference, cm (Control: n=18, Sit Less: n=17)** | | | | | |
| Control | 112.0 (105, 124) | 115.0 (103, 127) | 3.0 (2, 4) | 0.4 (-3.6, 4.4) | .86^b^ |
| Sit Less | 104.0 (95, 124) | 101.0 (96, 124) | 1.0 (0, 1) |  |  |
| **Waist/Hip Ratio (Control: n=18, Sit Less: n=17)** | | | | | |
| Control | 1.01 (0.9, 1.1) | 1.03 (0.8, 1.1) | 0.01 (-0.04, 0.03) | 0.02 (-0.04, 0.07) | .51^b^ |
| Sit Less | 0.98 (0.9, 1.01) | 0.97 (0.9, 1.1) | 0.00 (-0.03, 0.02) |  |  |
| **Systolic BP, mmHg (Control: n=18, Sit Less: n=17)** | | | | | |
| Control | 125.7 (115, 147) | 128.2 (111, 134) | -1.7 (-18, 6) | 0.6 (-5.7, 6.9) | .86^b^ |
| Sit Less | 129.7 (119, 139) | 125.7 (117, 132) | -4.7 (-9, -2)^c^ |  |  |
| **Diastolic BP, mmHg (Control: n=18, Sit Less: n=17)** | | | | | |
| Control | 75.8 (72, 84) | 70.8 (66, 90) | -7.8 (-10, 5) | -0.4 (-5.6, 4.9) | .91^b^ |
| Sit Less | 75.7 (71, 82) | 73.0 (69, 79) | -3.0 (-10, 2) |  |  |
| **Heart Rate, bpm (Control: n=18, Sit Less: n=17)** | | | | | |
| Control | 72.0 (63, 83) | 65.5 (61, 84) | -2.5 (-5, 2) | 3.6 (-3.0, 10.2) | .29^b^ |
| Sit Less | 66.0 (56, 73) | 61.0 (60, 71) | 0.0 (-5, 4) |  |  |
| **Insulin, µIU/mL (Control: n=14, Sit Less: n=14)** | | | | | |
| Control | 11.7 (8.9, 22.3) | 9.3 (5.7, 14.9) | -3.1 (-7.5, 2.2) | 1.4 (-7.9, 5.2) | .68^d^ |
| Sit Less | 11.0 (7.1, 17.2) | 9.0 (5.3, 15.2) | -1.6 (-3.9, 0.2) |  |  |
| **C-Reactive Protein, mg/L (Control: n=14, Sit Less: n=16)** | | | | | |
| Control | 2.5 (1.3, 7.6) | 1.9 (0.9, 5.3) | -0.6 (-1.7, -0.2)^c^ | 1.2 (-1.2, 3.5) | .32^d^ |
| Sit Less | 1.9 (0.5, 6.7) | 1.6 (0.8, 4.7) | 0.0 (-0.1, 0.3) |  |  |
| **Triglycerides, mg/dL (Control: n=17, Sit Less: n=14)** | | | | | |
| Control | 121.0 (87, 149) | 113.0 (101, 148) | 1.0 (-24, 13) | 24.4 (-23, 72) | .31^d^ |
| Sit Less | 110.0 (87, 134) | 89.0 (83, 133) | 7.0 (-30, 23) |  |  |
| **Total Cholesterol, mg/dL (Control: n=18, Sit Less: n=15)** | | | | | |
| Control | 139.0 (134, 174) | 148.5 (126, 165) | -3.0 (-11, -2)^c^ | -4.4 (-21, 12) | .60^b^ |
| Sit Less | 160.0 (136, 192) | 145.0 (125, 195) | -8.0 (-20, 4) |  |  |
| **HDL, mg/dL (Control: n=18, Sit Less: n=16)** | | | | | |
| Control | 32.5 (28, 38) | 38.0 (28, 43) | 1.0 (-6, 6) | 0.4 (-7, 8) | .91^d^ |
| Sit Less | 40.0 (35, 47) | 39.5 (30, 51) | -3.5 (-7, 9) |  |  |
| **LDL, mg/dL (Control: n=16, Sit Less: n=14)** | | | | | |
| Control | 80.5 (71, 108) | 72.0 (63, 93) | -7.0 (-21, 2) | -5.7 (-19, 7) | .38^b^ |
| Sit Less | 92.0 (79, 112) | 84.0 (70, 117) | -9.0 (-17, 6) |  |  |

Due to extreme skewness, median (IQR) and median change used to describe the observed distributions; All baseline differences were p > .05

^a^Adjusted for study cohort, cohort × group interaction, baseline value, and leisure-time exercise; estimates generated using robust standard errors

^b^ Test of the difference between adjusted marginal means using a generalized linear model with a Gaussian distribution and identity link, with robust standard errors (chi-square statistic, df=1).

^c^Within-group change statistically significant based on bootstrapped 95% CI

^d^ Test of the difference between adjusted marginal means using a generalized linear model with a Gamma test distribution, log link function, with robust standard errors (chi-square statistic, df=1).
